# SARS-CoV-2 Mutations and COVID-19 Clinical Outcome: Mutation Global Frequency Dynamics and Structural Modulation Hold the Key

**DOI:** 10.3389/fcimb.2022.868414

**Published:** 2022-03-21

**Authors:** Ranjeet Maurya, Pallavi Mishra, Aparna Swaminathan, Varsha Ravi, Sheeba Saifi, Akshay Kanakan, Priyanka Mehta, Priti Devi, Shaista Praveen, Sandeep Budhiraja, Bansidhar Tarai, Shimpa Sharma, Rajesh J. Khyalappa, Meghnad G. Joshi, Rajesh Pandey

**Affiliations:** ^1^ INtegrative GENomics of HOst-PathogEn (INGEN-HOPE) Laboratory, Council of Scientific & Industrial Research (CSIR)-Institute of Genomics and Integrative Biology (CSIR-IGIB), Delhi, India; ^2^ Academy of Scientific and Innovative Research (AcSIR), Ghaziabad, India; ^3^ Max Super Speciality Hospital (A Unit of Devki Devi Foundation), Max Healthcare, Delhi, India; ^4^ Dr. D. Y. Patil Medical College, Pune, India

**Keywords:** COVID-19, SARS-CoV-2, mutation analysis, disease outcome, global frequency flip, molecular dynamics simulation

## Abstract

Severe acute respiratory syndrome coronavirus 2 (SARS-CoV-2) has had an enormous burden on the healthcare system worldwide as a consequence of its new emerging variants of concern (VOCs) since late 2019. Elucidating viral genome characteristics and its influence on disease severity and clinical outcome has been one of the crucial aspects toward pandemic management. Genomic surveillance holds the key to identify the spectrum of mutations *vis-à-vis* disease outcome. Here, in our study, we performed a comprehensive analysis of the mutation distribution among the coronavirus disease 2019 (COVID-19) recovered and mortality patients. In addition to the clinical data analysis, the significant mutations within the two groups were analyzed for their global presence in an effort to understand the temporal dynamics of the mutations globally in comparison with our cohort. Interestingly, we found that all the mutations within the recovered patients showed significantly low global presence, indicating the possibility of regional pool of mutations and the absence of preferential selection by the virus during the course of the pandemic. In addition, we found the mutation S194L to have the most significant occurrence in the mortality group, suggesting its role toward a severe disease progression. Also, we discovered three mutations within the mortality patients with a high cohort and global distribution, which later became a part of variants of interest (VOIs)/VOCs, suggesting its significant role in enhancing viral characteristics. To understand the possible mechanism, we performed molecular dynamics (MD) simulations of nucleocapsid mutations, S194L and S194*, from the mortality and recovered patients, respectively, to examine its impacts on protein structure and stability. Importantly, we observed the mutation S194* within the recovered to be comparatively unstable, hence showing a low global frequency, as we observed. Thus, our study provides integrative insights about the clinical features, mutations significantly associated with the two different clinical outcomes, its global presence, and its possible effects at the structural level to understand the role of mutations in driving the COVID-19 pandemic.

## Introduction

The ongoing pandemic of severe acute respiratory syndrome coronavirus 2 (SARS-CoV-2) that emerged in late 2019 has had devastating impacts worldwide, and global collective efforts are being undertaken to characterize the virus through genomic surveillance and *in vitro* experiments (Mercatelli and Giorgi, 2020; Zhang et al., 2020). In comparison to other RNA viruses, SARS-CoV-2 has a lower evolutionary rate, although genetic mutations are one of the major mechanisms by which the virus evolves rapidly as a result of genetic selection. The evolving mutations, combined with large population size and short generation time, allow the virus to adapt to the host environment easily (Sanjuán and Domingo-Calap, 2016). Though most of the mutations induce null changes, a proportion of mutations are observed to relatively occur in higher incidences and cause challenges toward the development of vaccines, drugs, diagnostic tools, and even the disease clinical presentations (Lai et al., 2020).

Of all the mutations in different regions of the SARS-CoV-2 genome, the spike region is of major focus, as it facilitates the virus entry to the host *via* interaction with Angiotensin Converting Enzyme 2 (ACE2) receptors (Yang et al., 2020b). Several variants of interest (VOIs) and variants of concern (VOCs) with mutations in spike regions have been characterized and monitored to track the subsequent evolution of SARS-CoV-2 (https://www.who.int/en/activities/tracking-SARS-CoV-2-variants/). So far, several VOCs [B.1.1.7 (Alpha), B.1.351 (Beta), P.1 (Gamma), B.1.617.1 (Kappa), B.1.617.2 (Delta), B.1.525 (Eta), B.1.526 (Iota), C.37 (Lambda), B.1.621 (Mu), and presently B.1.1.529 and BA.1 and BA.2 (Omicron)] have been reported that modulate the viral transmission, disease severity, binding ability, and effectiveness of vaccines. One such VOC, Omicron, has more than 46 mutations in the spike protein with higher transmissibility and infectivity, leading to a global infection surge (Kannan et al., 2022). Due to the presence of an unusual number of mutations in the S protein, it might result in more efficient binding to ACE2 receptors and escape from neutralizing antibodies (Hu et al., 2022). However, a recent study demonstrated less efficient replication of Omicron due to its reduced dependence on Transmembrane Serine Protease 2 (TMPRSS2) for viral replication in comparison to Delta (Zhao et al., 2021). At the same time, data on disease severity are also steadily being reported from different parts of the world that would augment further understanding in this regard (https://www.who.int/en/activities/tracking-SARS-CoV-2-variants/).

The heterogeneity of clinical outcomes exhibited by SARS-CoV-2-affected individuals depends on numerous factors, inclusive of interindividual differences is the genotype-to-phenotype association for SARS-CoV-2 pathogenesis (Mousavizadeh and Ghasemi, 2021; Pereira et al., 2021). Several studies have reported high frequency mutation hotspots in SARS-CoV-2 genome regions that include ORF1a, S, and N (Kaushal et al., 2020). Majumdar and Niyogi (2020) reported P25L mutation in the Orf3a region to be strongly associated with higher mortality rates as a result of structural modification including the acquisition of escalated antigen diversity and B-cell epitope loss. Mutations in the NSP12 region that are involved in RNA-dependent RNA polymerase (RdRp) expression have been shown to contribute toward 5.9 times increased disease severity (Voss et al., 2020). Additionally, the mutation S194L in N protein was reported to be associated with the symptomatic patients and is located in the flexible linker region of the protein N (Zeng et al., 2020; Barona-Gómez et al., 2021).

Several mutations manifest as geographic patterns alluding to the virus ability to modify/adapt itself within a distinct microenvironment (Bakhshandeh et al., 2021). To acquire comprehension of these mutation patterns, it is important to have insights into their respective mutation frequency across both local and global patterns and help preempt the emergence of VOIs and VOCs (Justo Arevalo et al., 2021; Oude Munnink et al., 2021). Mutations along with host-modulating factors have been reported as a major contributor toward disease severity in COVID-19 (Mousavizadeh and Ghasemi, 2021; Pereira et al., 2021). Substitution of an amino acid residing in the antigen-determining region can possibly repress antibody-mediated immunity, resulting in enhanced virus proliferation (Gupta et al., 2020). Hence, it is crucial to investigate the correlation between mutations and patient clinical outcome that may play a role in SARS-CoV-2 pathophysiology and progression of disease severity (Toyoshima et al., 2020).

**Figure 1:**
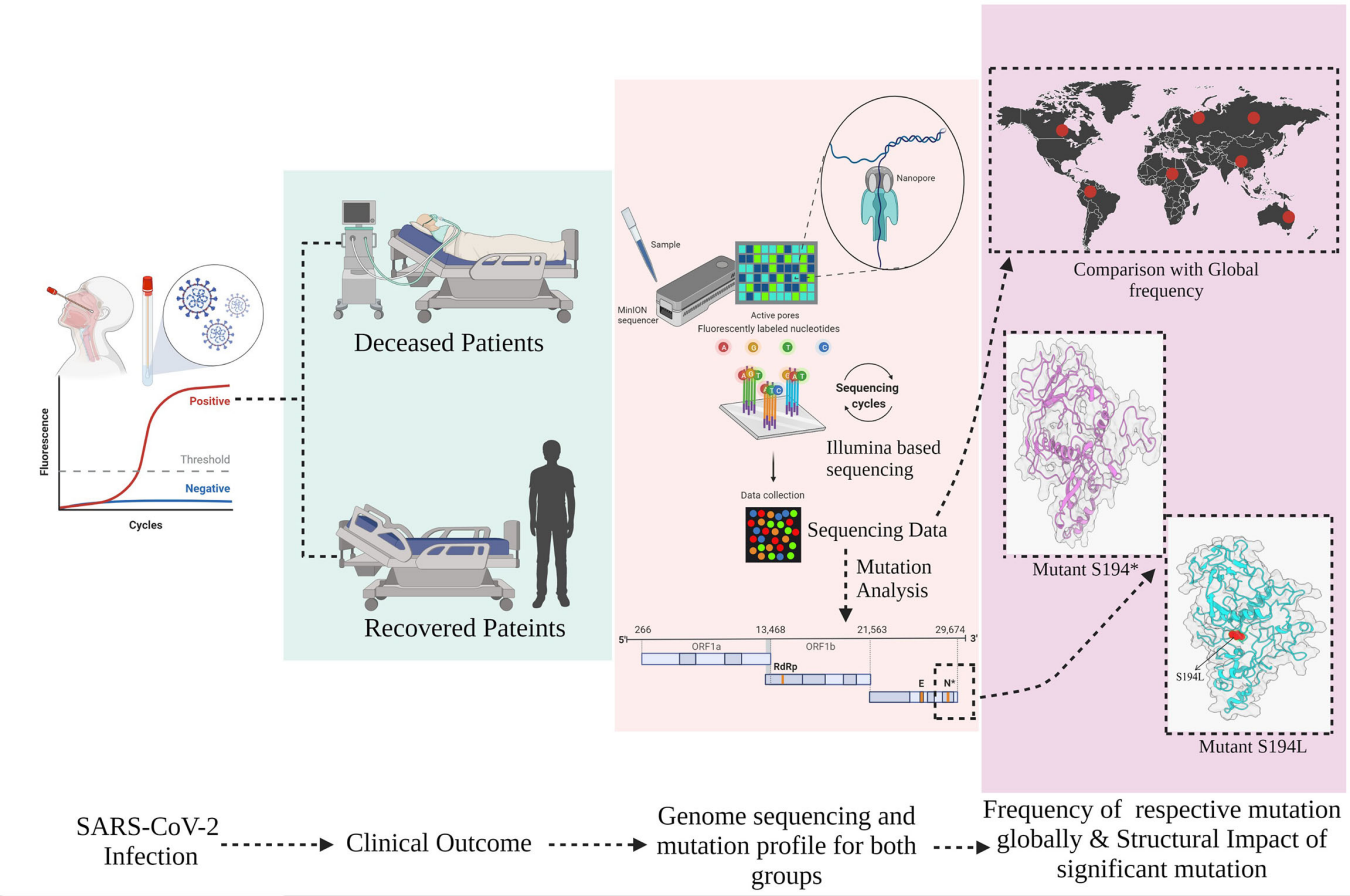
Overview of study design, stratification of hospitalized coronavirus disease 2019 (COVID-19) patients into *Recovered* and *Mortality*, and mutation prevalence across the cohort and global level including its structural consequences on the severe acute respiratory syndrome coronavirus 2 (SARS-CoV-2) protein.

The present study has sought to evaluate the mutational spectrum, clinical diversity, patient’s severity outcomes, and their respective global frequency in hospitalized COVID-19 patients. We further analyzed the structural modification of these mutations in SARS-CoV-2 that may alter its physiochemical properties leading to amelioration of pathogenicity (**Figure 1**).

## Methods

### Clinical Data and Classification

Clinical data of 246 COVID-19 patients were collected from Max Super Speciality Hospital, Delhi, and DY Patil Hospital, Maharashtra, India, and were assigned anonymous barcodes at CSIR-IGIB. The patients included in the study were confirmed SARS-CoV-2 positive by RT-PCR. The electronic medical records including patient demography, clinical symptoms, blood profile, comorbidities recorded at the time of admission, drugs administered to the patients during the course of infection were recorded and analyzed. The patients were classified into two major groups, *Recovered* and *Mortality*, based on the clinical outcome, as captured from the clinical data.

### RT-PCR

Viral RNA from VTM solutions was isolated using QIAmp viral mini kit, Qiagen, Cat. No. 52906, and SARS-CoV-2 detection and quantification were performed using TRUPCR SARS-CoV-2 kit (3B Black Bio Biotech India Ltd., Cat. No. 3B304) with a cycle threshold of 35. The detailed methodology has been published in the earlier publications from our lab.

### SARS-CoV-2 Whole-Genome Sequencing

Sequencing of the SARS-CoV-2 genomes was done using a combination of Oxford Nanopore Technology (ONT) and Illumina (MiSeq).

In brief, 100 ng of total RNA was used for double-stranded cDNA synthesis. This involves first-strand cDNA synthesis using Superscript IV (Thermo Fisher Scientific, Cat. No. 18091050), followed by RNase H digestion of ssRNA and second-strand synthesis by DNA polymerase-I large (Klenow) fragments (New England Biolabs, Cat. No. M0210S). Double-stranded DNA thus obtained was purified using 1.8× AMPure XP beads to sample ratio (Beckman Coulter, Cat. No. A63881). SARS-CoV-2 genome was then amplified from 100 ng of the purified cDNA with the ARCTIC V3 primer protocol. For sequencing library preparation using Oxford Nanopore sequencing, library preparation consists of End Repair/dA tailing, Native Barcode Ligation, and Adapter Ligation. This was performed with 200 ng of the multiplexed PCR amplicons according to ONT library preparation protocol-PCR tiling of COVID-19 virus (Version: PTC_9096_v109revE_06Feb2020). Sequencing in sets of 24 barcoded samples was performed on the MinION Mk1C platform.

Sequencing library preparation for Illumina was performed using 100 ng of purified ARTIC PCR product using the Illumina DNA Prep kit (Illumina, Cat. No. 20018705). The process involves tagmentation followed by post tagmentation cleanup and amplification by PCR, leading to indexed DNA fragments, which was purified prior to sequencing. The quality and quantity of the sequencing library were checked using an Agilent 2100 Bioanalyzer with high-sensitivity DNA chip and the Qubit dsDNA HS Assay kit, respectively. A loading concentration of 11 pM was prepared by denaturing and diluting the libraries in accordance with the MiSeq System Denature and Dilute Libraries Guide (Illumina, Document No. 15039740 v10). Sequencing was performed on the MiSeq system using the MiSeq Reagent Kit v3 (150 cycles) at 2 × 75 bps read length.

### Sequencing Data Analysis

The ARTIC end-to-end pipeline was used for the analysis of ONT MinION raw fast5 files up to variant calling. Raw fast5 files of samples were base called and demultiplexed using Guppy basecaller that uses the basecalling algorithms of Oxford Nanopore Technologies (Nanopore Community) with phred quality cutoff score >7 on GPU-linux-accelerated computing machine. Reads having Phred quality score less than 7 were discarded to filter the low-quality reads. The resultant demultiplexed fastq was normalized by read length of 300–500 (approximate size of amplicons) for further downstream analysis and aligned to the SARS-CoV-2 reference (MN908947.3) using the aligner Minimap2 v2.17 (Li, 2018). Nanopolish (Loman et al., 2015) was used to index raw fast5 files for variant calling from the minimap output files. To create consensus fasta, bcftools v1.8 was used with normalized minimap2 output.

For the Illumina sequencing data, FASTQC was performed for all the raw fastq files generated to check the Phred quality score of all the sequences (FastQC A Quality Control tool for High Throughput Sequence Data, Babraham Bioinformatics). A phred quality score threshold of >20 was used for filtering reads from all the samples. Next, adapter trimming was performed using Trim Galore tool (–TrimGalore, Babraham Bioinformatics) and alignment of the sequences was performed using HISAT2 algorithm (Kim et al., 2019) on human data build hg38 to remove any human read contamination. BEDTools was used to generate the consensus fasta using the unaligned/filtered reads, and variant calling was performed using the high-quality reads (Quinlan and Hall, 2010).

### Phylogenetic Analysis

The Wuhan reference genome for SARS-CoV-2 (NC_045512.2) was used to perform multiple sequence alignment using MAFFT (v7.475) (Katoh et al., 2002). The alignment was manually trimmed, and a phylogenetic tree was constructed using the IQ-tree (Nguyen et al., 2015). Lineage classification was performed using PANGOLIN. The phylogenetic analysis was visualized using FIGTREE software (http://tree.bio.ed.ac.uk/software/figtree/).

### Mutation Analysis

The vcf files were used to get the frequency of the mutations in the samples for the mutational spectra analysis within the recovered and mortality patients, and snpEff v5.0 (Cingolani et al., 2012) has been used to perform the variant annotation information such as the variant definition and effect of the variants. SnpEff database has been created by “SnpEff build” using the Wuhan reference NC_045512.2. Furthermore, global frequency of mutations was checked against a global dataset available at 2019 Novel Coronavirus Resource (2019nCoVR), China National Center for Bioinformation (CNCB) (Song et al., 2020). The lineages defining mutations for different VOCs/VOIs were curated from two public databases (https://covariants.org/shared-mutations, https://github.com/cov-lineages/constellations). A comparative study of mutation frequency in recovered and mortality patients with the respective cumulative global frequency of mutations plotted as the lollipop plot representing the mutations was generated in R v4.1.0 using g3viz (Guo et al., 2020), rtracklayer (Lawrence et al., 2009), and trackViewer (Ou and Zhu, 2019) packages followed by data visualization using the ggplot2 (R package ggplot2 v3.3.5) package. Variant position along the SARS-CoV-2 genome is indicated in the plot, which is used to show the frequent mutations with the global frequency, which is later selected as VOC/VOI.

### Quantification and Statistical Analysis

The statistical analysis was performed by R software. The nonparametric Fisher exact test of significance for independence between two categorical variables was performed to check the difference of mutation profile for the mortality vs. recovered patients. All *p*-values were calculated from 2-sided tests using 0.05 as the significance level. The direction of the association between mutation and group was calculated using phi-coefficient correlation (r_φ_) by measuring the strength of association.

### Generation of Wild-Type and Mutant N-Protein Models

MD was performed on N protein (uniprot id-P0DTC9), with fasta sequence retrieved from the UniProt database and the protein being modeled using Phyre (http://www.sbg.bio.ic.ac.uk/phyre2/html/page.cgi?id=index). The mutation for recovered (S194*) and mortality (S194L) was incorporated using PyMOL (PyMOL | pymol.org). The protein complex simulation is done with 200 ns of timescale for understanding the dynamic behavior and the interaction pattern (Shafreen et al., 2013; Selvaraj et al., 2018). Structures are analyzed using the CHARM-AA force field solvated by the SPC16 water model within a periodic boundary box of distance 1.0 nm, fixed between the protein and cubic box (Nguyen et al., 2014; Bandaru et al., 2017). For neutralizing the whole system, the accurate concentration of (Na^+^/Cl^−^) ions is added based on the rebalancing charges. Initial energy minimization is appended with the prepared complex systems for 1,000 steps of steepest descent algorithm *via* a tolerance of 10 kJ/mol/nm to avoid the steric clashes (Kumar et al., 2021). Thermostat coupling is set with a reference temperature of 300K using Berendsen thermostat and pressure coupling with 1.0 bar reference pressure using Parrinello-Rahman along with periodic boundary conditions with cutoffs for Lennard-Jones and Coulomb interactions. The particle mesh Ewald method is used for calculating the long-range interactions for biomolecular systems (Wohlert and Edholm, 2004).

After the initial minimization step, the whole system is again well equilibrated at 2,000 ps at 300K and 1 bar pressure in Number of particles system volume and temperature (NVT) and Number of particles system pressure and temperature (NPT) ensembles (Childers and Daggett, 2018). Final MD simulation step is processed for three protein complexes (wild-type, recovered, and mortality N-proteins) with respect to the timescale of 200 ns. For MD simulation analysis, the results of root mean square deviation (RMSD) and root mean square fluctuation (RMSF) are performed using the XMGRACE v5.1.2 and GROMACS v.5.1.2 tools.

## Results

### Patient Clinical Demographics and Classification

**Figure 2:**
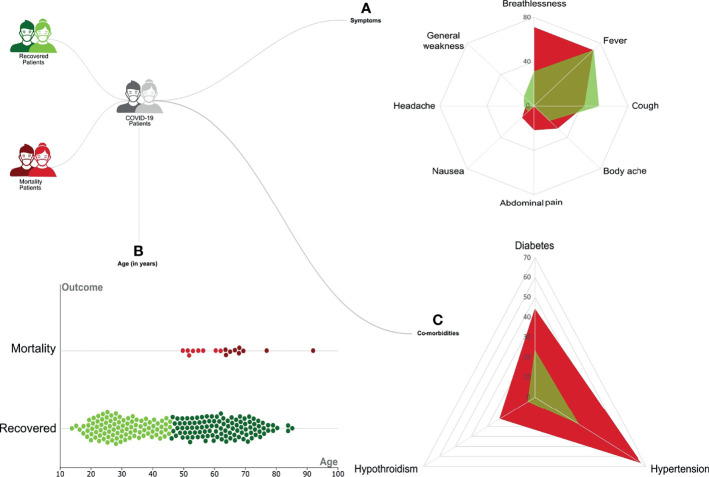
Representation of the clinical demographics of the coronavirus disease 2019 (COVID-19) patients. A total of 246 COVID-19 patients were divided into 228 *Recovered* (in green) and 18 *Mortality* (in red). The categorical clinical features presented by the patients are as follows: **(A)** Differential presence of various symptoms like breathlessness, fever, cough, body ache, nausea, headache, and general weakness between the *Recovered* (in green) and *Mortality* (in red). **(B)** The age of patients in the two groups is represented as a violin plot where the darker color represents the upper quartile range. **(C)** Differential presence of comorbidities like diabetes, hypertension, and hypothyroidism between the *Recovered* (in green) and *Mortality* (in red).

Our initial dataset was curated to exclude patients with any missing data for important parameters, resulting in a total sample size of 246 patients. These patients were classified based on the clinical outcome, with 228 *Recovered* and 18 *Mortality* patients. Regression analysis using the chi-square test and Kruskal–Wallis test highlighted a significant distribution of specific clinical parameters. Symptoms such as shortness of breath (*p*-value = 0.037) and abdominal pain (*p*-value = 0.001) were observed to be significantly associated with the mortality patients (**Figure 2A**). Among the patients with comorbidities, hypertension (*p*-value = 0.01) and hypothyroidism (*p*-value = 0.01) were significantly correlated with the mortality (**Figure 2C**). Notably, most of the patients who recovered did not significantly present any comorbidities (*p*-value = 0.001). Also, a difference in age was seen among the recovered and mortality patients (*p*-value = 0.01) (**Figure 2B**). All the mortality patients were on respiratory support, whereas only 28% those recovered required respiratory support. The clinical parameters and analysis are shown in **Table 1**.

**Table 1:** Clinical summary of the COVID-19 patients highlighting clinical parameters across *recovered* and *mortality*.

Groups	Total (n = 246)	Mortality (n = 18)	Recovered (n = 228)	*p*-value
Gender F|M	79|166	3|14*	76|152	0.18^b^
Age	48 (30–63)	63 (55–68)	46 (30–63)	**<0.001****^a^**
Respiratory Support	83 (33.73%)	18 (100%)	65 (28.50%)	–
E gene	24.19 (21.21–27.51)	22.48 (19.62–27.69)	24.34 (21.56–27.51)	0.21^a^
RdRp gene	25.11 (21.51–28.91)	23.67 (20.59–28)	25.24 (21.57–28.94)	0.26^a^
**Symptoms**				
Shortness of Breath	82 (33.33%)	10 (55.56%)	72 (31.57%)	**0.037****^b^**
Fever	174 (70.73%)	10 (55.56%)	164 (71.92%)	0.141^b^
Sore Throat	132 (53.65%)	6 (33.33%)	126 (55.26%)	0.72^b^
Body ache	47 (19.10%)	4 (22.22%)	43 (18.85%)	0.72^b^
Abdominal pain	4 (1.62%)	3 (16.67%)	1 (0.43%)	**<0.001****^b^**
Nausea	18 (7.31%)	2 (11.11%)	16 (7.01%)	0.52^b^
Headache	20 (8.13%)	1 (5.56%)	19 (8.33%)	0.64^b^
General weakness	28 (11.38%)	0 (0)	28 (12.28%)	–
No Co-morbidities	141 (57.31%)	4 (22.22%)	137 (60.08%)	**<0.001****^b^**
**Comorbidities**	105 (42.68%)	14 (77.77%)	91 (39.92%)	
Diabetes	62 (25.20%)	8 (44.44%)	54 (23.68%)	0.05^b^
Hypertension	74 (30.08%)	12 (66.66%)	62 (27.19%)	**<0.001****^b^**
Hypothyroidism	14 (5.69%)	4 (22.22%)	10 (4.38%)	**<0.001****^b^**
Asthma	4 (1.62%)	0 (0%)	4 (1.75%)	–
Hospital stay	11 (5–14)	13 (6–18)	11 (5–14)	0.34^a^

Data are shown as median [interquartile range (IQR)] or n (%).

a Kruskal–Wallis test.

b Chi-square test.

*Missing data.

Values of significance are highlighted in bold.

### SARS-CoV-2 Phylogenetic Analysis and Distribution

**Figure 3:**
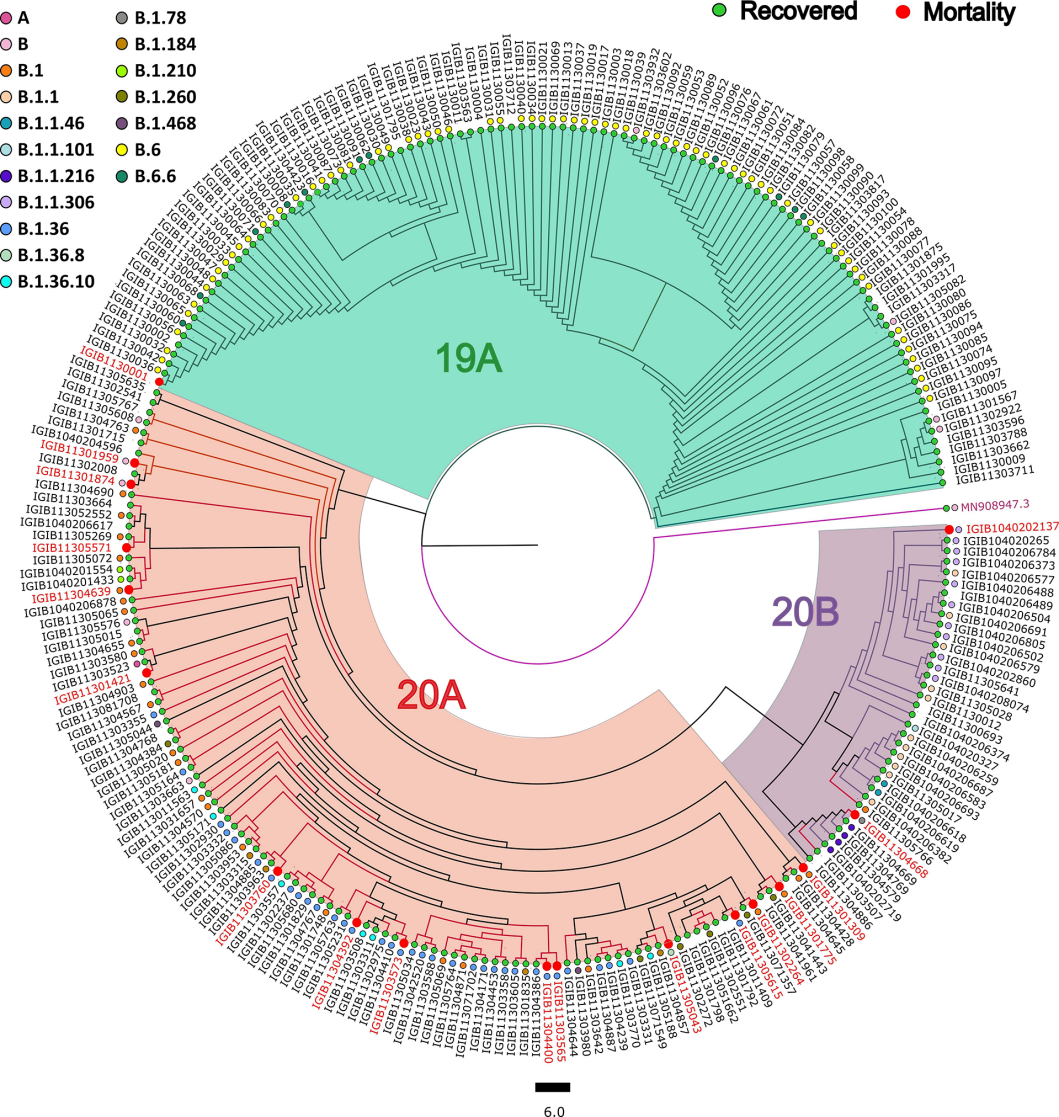
Phylogenetic analysis of the severe acute respiratory syndrome coronavirus 2 (SARS-CoV-2) genomes. Showing the distribution of 246 SARS-CoV-2 clades and lineages among COVID-19 patients compared with the wild-type strain.

Phylogenetic analysis of 246 SARS-CoV-2 genomes using PANGOLIN (lineage) and Nextstrain (clades) showed the dominant occurrence of B.6 (29.2%), B.1.36 (9.82%), B.1 (10.56%), B.1.1 (4.47%), B.1.1.306 (4.47%), and B.6.6 (4.47%), whereas clade distribution highlighted the dominant prevalence of 19A (52.84%) followed by 20A (34.55%), as mentioned in **Supplementary Table S1**. We observed that the least number of patients belonged to the 20B (13%) clade, whereas 19A has the highest number of patients. Delving further, within the recovered samples, major distribution for the lineage was B.6 (31.57%), B.1.36 (12.28%), and B.1 (9.64%), whereas 19A was the dominant clade (45.17%); and within mortality, major distribution for the lineages was B.1.36 (33.33%) followed by B.1 (22.22%) and 20A clade (83.33%) (**Figure 3**).

### SARS-CoV-2 Mutation Profile Within Different Clinical Outcomes and Its Frequency Flip

**Figure 4:**
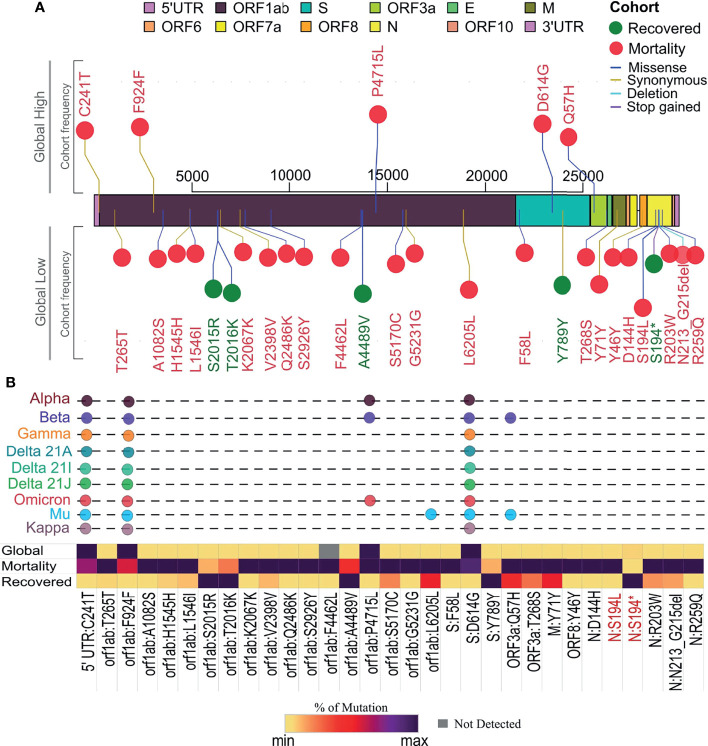
Segregation of mutation profile across recovered and mortality coronavirus disease 2019 (COVID-19) patients. **(A)** Mutations with significant association with recovered (green) and mortality (red) patients along the SARS-CoV-2 genome and global presence. **(B)** Showing percentage distribution of mutations in the two clinical groups, their global frequency, and their presence in existing variants of interest (VOIs) and variants of concern (VOCs) as signature mutations.

Individual mutation analysis across our sample cohort revealed a total of 3,449 SARS-CoV-2 genome mutations. Importantly, we observed that within the mortality cases in our cohort, there was a higher number of mutations per sample, 28.94, than the recovered group, which had 13.60 mutations/sample. This observation made us explore the possible reasons for higher mutations per sample within mortality and their role in disease outcome. The Fisher exact test that was performed across our cohort revealed a total of 31 mutations to be statistically significant, with a *p*-value <0.005 associated with the clinical outcome (**Supplementary Table S2**, **Figure 4A**). Further correlation analysis of these 31 mutations resulted in the stratification of five mutations into the recovered and 26 mutations with the mortality patients. When compared with their global frequency, 7/31 significant mutations showed a low global presence indicating a frequency flip from their high distribution observed in our samples. The segregation of these seven mutations exhibited frequency flips with five mutations within the recovered group and two mutations in the mortality group, showing a significant difference in their association with the respective groups (*p*-value = 0.0001). Notably, all the mutations associated with the recovered group (S2015R, Y789Y, T2016K, S194*, A4489V) showed a frequency flip, whereas two mutations associated with the mortality group showed a shift in their frequency from our cohort to global level. The recovered group displaying a frequency flip for all the mutations could indicate that these mutations are highly likely to be deselected by the virus during the course of its evolution. On the other hand, among the rest of the mutations in the mortality group that did not exhibit any frequency flip (24 mutations), we observed five mutations (C241T, F924F, P4715L, D614G, Q75H) with a high cohort and global distribution. Interestingly, all these five mutations subsequently became a part of VOIs/VOCs such as B.1.1.7 (Alpha), B.1.351 (Beta), P.1 (Gamma), B.1.617.1 (Kappa), B.1.617.2 (Delta), B.1.525 (Eta), B.1.526 (Iota), C.37 (Lambda), B.1.621 (Mu), and presently being seen in BA.1 and BA.2 (Omicron) (**Figure 4B**). Hence, the presence of these mutations at high frequency throughout the pandemic and its selection in VOIs/VOCs could possibly suggest the propensity of the virus to retain these mutations, thereby enhancing the functional characteristics of SARS-CoV-2.

### Analysis of Molecular Dynamics Trajectories for Effect of Mutation on the Protein Structure

**Figure 5:**
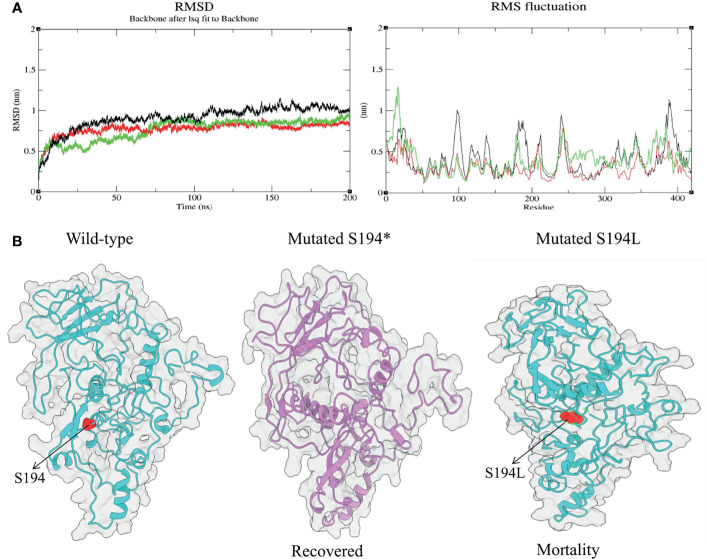
Structural changes during molecular dynamic simulation: **(A)** Superimposed root mean square deviation (RMSD) and root mean square fluctuation (RMSF) spectrum of wild-type (black) and mutant proteins [S194* (recovered) shown in green and S194L (mortality) shown in red] during 200 ns of molecular dynamics simulation period. **(B)** The obtained simulated structures of mutated N protein with wild type.

Among the significant mutations, we found S194L in the N protein to be highly significant (*p*-value = 2.35481E-13) and exclusive to the mortality patients. Similarly, a mutation at the same position, S194*, in the N protein was observed in the recovered as well, which was unique and significant with a *p*-value of 0.0045. Evidently, N protein is reported to be more conserved in comparison to other proteins such as Spike and membrane glycoprotein, which plays an essential role in viral genome packaging (Cubuk et al., 2021). Henceforth, we performed MD simulations to study and analyze the consequences of these two mutations compared to the wild-type N protein, in an endeavor to understand its association with the disease outcome. The RMSD was calculated for wild-type and mutants to elucidate changes in the overall stability of the protein that was considered the primary criterion for calculating the overall convergence of the system. The deviations for all the three systems (wild-type, S194*, and S194L) were found to be between 0.1 and 1.1 nm (**Supplementary Table S3**). A state of equilibrium was achieved for both the mutant proteins at 115 ns; whereas for the wild-type, it was at 150 ns. Subsequently, we calculated the fluctuations of the residues using RMSF (**Figure 5A**). There were local changes observed, with higher peaks of fluctuations seen at 10–18 and 179th positions in the case of S194*, reaching up to 0.8–1.2 nm and 0.7 nm, respectively (**Supplementary Table S4**). Fluctuation was seen more in N protein with S194* as compared to the S194L mutant N protein (**Figure 5B**). It is indicative of the recovered patients exhibiting a comparatively unstable protein *vis-à-vis* the mortality patients.

## Discussion

There is a significant amount of SARS-CoV-2 genome sequencing data being deposited in public databases in real time. However, our comprehension of different patient clinical presentations and their correlation with the SARS-CoV-2 mutations remains limited (Esper et al., 2021). Toward that, we made an effort to analyze and correlate the SARS-CoV-2 mutations within the recovered and mortality patients along with their impacts on the protein structure. Clinically, similar to other studies from around the globe, advanced age and underlying conditions such as hypertension and hypothyroidism were significantly associated with the mortality of COVID-19 patients (Wang et al., 2020; Zhou et al., 2020; Huang et al., 2021; Li et al., 2021). With reference to presenting symptoms as potential predictors for disease severity, a rare symptom, abdominal pain, was also observed to be significantly associated with the mortality group as reported by Li et al. (2021).

Genomic analysis of 246 samples demonstrated that the lineage B.6 had a dominant occurrence followed by B.1.36, B.1, B.1.1, B.1.1.306, B.6.6, and 20A for the clade, respectively, which is consistent with the lineage distribution observed during the first wave in India (Chong et al., 2020; Jha et al., 2021). Moreover, a significant frequency flip (*p*-value = 0.0001) was seen for the five mutations, S2015R, Y789Y, T2016K, S194*, and A4489V, in the recovered with a low global presence, indicating that the mutations associated with COVID-19 recovery are deselected by the virus during its evolution. Intriguingly, 5/26 significant mutations (C241T, Q57H, F924F, P4715L, D614G) in the mortality patients did not exhibit any frequency flip with a high frequency occurrence observed in the cohort as well as the global level. This could be indicative that the mutations associated with severe outcome are preferably selected and present globally. A study by Mercatelli and Giorgi (2020) also demonstrated the mutations C241T, F924F, and P4715L to have a similar frequency as D614G in their cohort as well, which shows their co-occurrence in the viral genome.

It is interesting that these five mutations are the characteristic mutations of Type VI SARS-CoV-2 strain and show a high pairwise allelic association. Moreover, the mutation P4715L in RdRp protein affects the viral replication (Pachetti et al., 2020), and the spike mutation D614G influences the viral interaction with the ACE2 receptor, thereby enhancing the overall fitness of the virus (Korber et al., 2020; Toyoshima et al., 2020). Also, the Type VI strain is observed to be present in high frequency in most of the countries throughout the pandemic that could possibly imply that these mutations provide stability and positively influence the virulence by enhancing the viral transmission and pathogenicity (Yang et al., 2020a). A study by Bianchi et al. (2021) reported that the mutation Q57H, which is observed in our mortality patients, is consistently present in high frequency at all the time points. Another study describes the loss of function of orf3b protein, an accessory protein, due to the truncation of orf3a protein by Q57H mutation. Furthermore, they also found that the loss of orf3b coincides with the emergence of D614G spike mutation that strikingly correlates with our observation of D614G being present at high frequency in our mortality patients (Lam et al., 2020). Also, the mutation S194L found in the Nucleocapsid gene was a highly significant mutation observed across our cohort and is also associated with mortality. Several other studies have also reported that this mutation is deleterious and correlated with fatal outcomes (Limaye et al., 2021; Mehta et al., 2021; SeyedAlinaghi et al., 2021). This could possibly indicate their crucial role in elevating the pathogenicity of the virus, thereby contributing to increased disease severity and clinical outcome. Such significant observation can aid us in tracking and prioritizing the upcoming SARS-CoV-2 variants as well as monitoring the disease progression for early interventions to possibly manage fatal outcomes.

To understand the correlation of mutations with severity outcome, one of the important aspects is its functional consequences at the protein structural level. The MD simulations performed for the mutations S194* and S194L from the recovered and mortality group, respectively, revealed RMSD values, which was evaluated for both the mutations and the wild-type, implying a state of equilibrium achieved for all the three systems. Although mutation S194L is previously reported to be associated with mortality as seen in our cohort, its effect on the protein structure has not been explored and elucidated (Joshi et al., 2021). A comparative analysis of the parameter RMSF value demonstrated that the recovered mutant protein S194* showed more fluctuations in comparison with the wild type and the protein mutation at the same position associated with the mortality group. This suggests the instability of the mutation S194* in the recovered group, which is exactly correlating with its low frequency occurrence at the global level as compared to S194L in the mortality group.

In summary, the findings from our study highlight the significance of the mutations in modulating COVID-19 presentations and outcomes. Assessing the clinical symptoms of COVID19 patients is largely important, since patients manifest a wide spectrum of symptoms, from asymptomatic to symptomatic and a subset leading to fatal outcomes. Disease symptoms and its progression are modulated by several factors that include viral characteristics, environmental factors, host immunity, and comorbidities. An integrative analysis of these disease modulators can aid us in devising strategies for the disease symptom management and treatment, if possible. The co-analysis of mutations and the clinical outcome would be extremely beneficial for diagnosing mutations susceptible to increased disease severity and also to evaluate the effects of control measures implemented during the course of the pandemic. This approach can assist us in tracking the SARS-CoV-2 evolution and its upcoming variants, along with providing insights to its association with the disease severity or outcome for the amelioration of the disease. Thus, we can identify the subgroups of patients predicted to have a poor prognosis and can be assisted with required treatments prior to any detrimental outcomes. Hence, this approach can lead to a better utilization of the healthcare management system and could aid us in a sustainable pandemic preparedness.

## Data Availability Statement

The clinical dataset collected and analyzed as part of this study is available in the **Supplementary Material** as **Data Sheet 1**. The SARS-CoV-2 genomic data have been uploaded to GISAID.

## Ethics Statement

The studies involving human participants were reviewed and approved by CSIR-IGIB’s Human Ethics Committee Clearance (Ref No: CSIR-IGIB/IHEC/2020-21/01). The patients/participants provided their written informed consent to participate in this study.

## Author Contributions

RM, PM, VR, AK and PrM performed the analysis. RM, PM, AS, VR, SS, AK, PrM, PD, SP and RP wrote the article. RP designed, conceptualized, implemented and coordinated the study. SB, BT, ShS, RJK and MGJ shared the clinical samples and clinical data. All authors contributed to the article and approved the final submission.

## Funding

This research was funded by the Bill and Melinda Gates Foundation (BMGF) [Project code : INV-033578], Indo-US Science and Technology Forum (IUSSTF) [Project code : CLP-0033], Foundation for Innovative New Diagnostics (FIND) [Project code : CLP-0038], and AIDS Healthcare Foundation (AHF) [Project code : CLP-0043].

## Conflict of Interest

The authors declare that the research was conducted in the absence of any commercial or financial relationships that could be construed as a potential conflict of interest.

## Publisher’s Note

All claims expressed in this article are solely those of the authors and do not necessarily represent those of their affiliated organizations, or those of the publisher, the editors and the reviewers. Any product that may be evaluated in this article, or claim that may be made by its manufacturer, is not guaranteed or endorsed by the publisher.

## Supplementary Material

Supplementary Table 1 Distribution of clade and lineages across 246 cohort samples.

Supplementary Table 2 Individual mutations across our sample cohort with its statistical significance (*p-value*) and phi-coefficient correlation (*rφ*) value.

Supplementary Table 3 Results of Root mean square deviation (RMSD) during simulation analysis with respect to the timescale of 200ns.

Supplementary Table 4 Results of Root mean square fluctuation (RMSF) during simulation analysis with respect to the timescale of 200ns.

Supplementary Data Sheet 1 GISAID Accession numbers associated with samples used in the Cohort.
